# Between Canalization and Plasticity: The Role of Obsessive–Compulsive Rituals in Evo-Devo Psychopathology

**DOI:** 10.3390/brainsci16020164

**Published:** 2026-01-30

**Authors:** Matteo Tonna

**Affiliations:** 1 Psychiatric Unit, Department of Medicine and Surgery, University of Parma, 43121 Parma, Italy; matteo.tonna@unipr.it; 2 Department of Mental Health, Local Health Service, 43121 Parma, Italy

Current research highlights the clinical heterogeneity of obsessive–compulsive disorder (OCD) and, particularly, of OCD rituals, which are engrained in a variety of developmental pathways, psychopathological vulnerabilities, internal perturbations and external precipitating factors (Tonna, 2024; contribution 1). However, to date there have been few attempts to understand OCD rituals from a trans-diagnostic and interdisciplinary perspective.

Rituals show a strikingly long evolutionary history, at least dating back to vertebrate phylogeny, and, consistently, present a highly evolutionary-conserved motor structure, wired in basal ganglia circuitry. Their fixed behavioral pattern, built upon the fragmentation of the action flow through the repetition of acts (functional acts) and/or the insertion of acts “out of context” (non-functional acts), evolved from pre-programmed motor responses (e.g., fixed-action patterns) and habitual behaviors to cope with conditions of unpredictability. In fact, repetition of acts is ubiquitous behavioral patterns in the build-up of the motor repertoire, whereas non-functional acts contribute to re-aligning behavior with changing environments. Ultimately, these motor strategies are involved in behavioral adjustment over any ecological disorders, perturbations and “high-entropy” states [1,2].

The key concept is that in humans, the problem of facing potential behavior–environment mismatches is hugely emphasized, especially in the developmental years, and, as such, the recourse to rituals. In fact, during human speciation, brain connectivity underwent increasing neural flexibility to be exploited/redeployed/readapted for novel functions, such as higher cognitive/social skills and language, within complex biocultural reverberating cycles [3]. During development this high flexibility is mirrored by a sensorimotor repatterning pushed towards increasingly unconstrained neural configurations by extensive neotenic synaptic plasticity to host emergent domains within it. Closing the loop, psycho-socio-cultural feedback mechanisms play a pivotal role in the developmental fine-tuning of such “relaxed” sensorimotor networks to their eco-cultural niches.

Sensorimotor features of relaxation and openness, however, come at the cost of a broad spectrum of phenotypic variation (i.e., reaction norms) under different environmental pressures [4], potentially leading to relevant deviations in normative developmental trajectories [5]. Consistently, recently evolved systems are more susceptible to reaction norms [6]. In this connection, the reuse of sensorimotor networks for evolutionarily novel functions makes the sensorimotor system more vulnerable to divergent developmental pathways. Therefore, sensorimotor repatterning is “canalized” to prevent adverse developmental impacts on the system itself. Canalization refers to the tendency of developmental processes to follow invariant trajectories, despite external or internal disturbances. In this vein, many developmental pathways should be better conceived as dynamic systems with “homeorhetic” features, i.e., with the property to return to a particular trajectory under environmental pressures [7]. Canalization and homeorhesis are inherent features of the sensorimotor system, in order to finely balancing the evolutionary trade-off between pre-existing functions (e.g., multisensory and sensorimotor integration processing) and novel functions (e.g., thought abstraction and language processing) and thus, between neural constraints (for internal stability and efficiency of the system) and neural plasticity (for emergent demands within the system).

In this regard, we propose that OCD rituals may play a pivotal role in mechanisms aimed at stabilizing canalized trajectories under various developmental pressures. In fact, many recent contributions all emphasize from different perspectives the complex interplay between deviations from normative development, internal or environmental perturbations, and OCD rituals. Particularly, the recourse to ritual behavior seems to be associated with different exogenous or endogenous factors, which ultimately have an impact on neurodevelopment, specifically on sensorimotor maturation. In this vein, Tal and colleagues (2023; contribution 2) point to a strong association between atypical sensory (exteroceptive and interoceptive) processing and ritual compulsions. Coherently, obsessive–compulsive symptoms show a widespread synchronic and diachronic comorbidity with conditions that affect the sensorimotor system, from neurodevelopmental disorders (e.g., autism spectrum) (see for example: Aymerich et al., 2024; contribution 3; Dell’Osso et al., 2024; contribution 4) to early traumatic experiences and schizophrenia vulnerability (Borrelli et al., 2024; contribution 5); moreover, OCD rituals are associated with both internal perturbations (e.g., gut microbiome alterations and inflammatory bowel disease) (Grassi and Pampaloni, 2024; contribution 6) and external, psycho-social injuries (such as COVID-19 pandemic) (D’Urso et al., 2024; contribution 7). Remarkably, Gambolo’ and colleagues (2025; contribution 8) suggest that the motor structure of ritual itself may reflect a specific developmental background, as rituals primarily built upon acts repetition are more associated with both pre-psychotic symptoms and childhood trauma than those mainly based on non-functional acts.

This is not to say that the multifaceted clinical presentation of OCD may be reduced to its motor underpinnings. Actually, an evolutionary developmental (evo-devo) approach encourages an inter-mixing between “bottom-up” and “top-down” models. During phylogeny and ontogeny, in fact, motor patterns become progressively intertwined with specific cognitive and metacognitive styles (e.g., peculiar memory dysfunctions), which recursively contribute to reinforce and sustain compulsive behavior (Gurrieri et al., 2025; contribution 9). On the other hand, the pathophysiology of OCD rituals is centered in the cortico-striato-thalamo-cortical (CSTC) circuitry, which, due to its long phylogenetic history, is involved in different functional networks, and equally participates in behavioral, emotional, cognitive and social processes [8].

Overall, contemporary research suggests a complex interaction between genetic and environmental factors on the neuroplasticity of a developing brain in modulating a bunch of behavioral outputs, such as OCD rituals and compulsive-like behaviors (Huang et al. 2025; contribution 10). Particularly, in humans, obsessive–compulsive rituals seem to be over-expressed whenever internal or external perturbations impact developmental trajectories, ultimately converging into disordered sensory processing. As such, rituals would follow their phylogenetic causations, by coping with any condition of perceptual unpredictability. In this regard, they would impose order and stability on the developing sensorimotor system, in an effort to re-align the canalized trajectories and minimize the effect of co-occurring disrupting factors. Coherently, growing evidence indicates that obsessive–compulsive symptoms have an impact on the levels of entropy in different brain areas [9]. In dynamic systems, entropy is an index of the internal complexity, randomness and unpredictability of the system (measured via fMRI or EEG). Therefore, obsessive–compulsive symptoms could play a role in the balancing between stability/predictability and flexibility/unpredictability of brain networks, the effect of which varies depending on the specific sub-system considered [10].

From this perspective, rituals should be conceived as adaptive, evolved behavioral traits, which may vary among the population, continuously grading between the two extremes of under-expression and over-expression, as many other biologic, cognitive and behavioral functions and systems [11]. In this vein, OCD rituals represent the over-expressed extreme of such a variation or, to put it differently, its psychopathological counterpart. First and foremost, they are not always due to a primary dysregulation of the behavioral system in question, but, particularly in the developmental years, they may be triggered, upon a genetic predisposition, as adaptation responses to other psychopathological vulnerabilities or bio-psycho-social mismatches, representing their early biomarkers. In this latter condition, the underlying developmental perturbation pushes the average variation of the trait towards the over-expressed end of the continuum, probably through epigenetic mechanisms [12].

Under an evo-devo perspective, we can fully capture the ultimate causes (e.g., the phylogenetic origin and adaptive significance) and the proximate mechanisms (e.g., the functional and structural underpinnings) of OCD rituals as well as their widespread comorbidity in psychopathology, rather than considering them merely in terms of symptoms or deficits. As such, this approach may provide new insights on the complex interaction between ritual behavior and canalized trajectories in developing systems, which are inherently dynamic and plastic. Particularly, the contributions included in this Editorial hint at a central role of obsessive–compulsive rituals in the fine balancing between constraints and flexibility, canalization and plasticity, perturbations and compensations, deviations and realignments during development. Finally, it is intriguing to hypothesize a functional continuity for ritual behavior in the emergent dynamics which marked human speciation; particularly, the ordering and stabilizing effect of rituals on eco-cultural constructed niches may have scaffolded the co-evolutionary feedback loops among increasingly complex societies, brain enlargement and the development of symbolic language.

Future research should be addressed in elucidating the epigenetic mechanisms beneath the persistence of OCD rituals after early infancy (where they constitute a normative behavior). Moreover, the specific role played by rituals in different neurodevelopmental trajectories is far from being understood. Particularly, rituals’ proximate causations over sensorimotor atypies should be better investigated. Longitudinal studies are needed to prospectively evaluate the impact of ritual behavior on specific developmental pathways, and its possible pathoplastic effect on the phenotypic expression, course and outcome of different underlying psychopathological vulnerabilities. Finally, research on OCD rituals would benefit from an inter-disciplinary approach, capable of embracing different perspectives (ethology, neuroscience, psychopathology, social sciences and anthropology), within a shared evo-devo framework.
